# Targeting Germ Cell Tumors with the Newly Synthesized Flavanone-Derived Compound MLo1302 Efficiently Reduces Tumor Cell Viability and Induces Apoptosis and Cell Cycle Arrest

**DOI:** 10.3390/pharmaceutics13010073

**Published:** 2021-01-07

**Authors:** João Lobo, Ana Rita Cardoso, Vera Miranda-Gonçalves, Leendert H. J. Looijenga, Marie Lopez, Paola B. Arimondo, Rui Henrique, Carmen Jerónimo

**Affiliations:** 1 Cancer Biology and Epigenetics Group, IPO Porto Research Center (GEBC CI-IPOP), Portuguese Oncology Institute of Porto (IPO Porto) & Porto Comprehensive Cancer Center (P.CCC), R. Dr. António Bernardino de Almeida, 4200-072 Porto, Portugal; jpedro.lobo@ipoporto.min-saude.pt (J.L.); ana.almeida.cardoso@ipoporto.min-saude.pt (A.R.C.); vera.miranda.goncalves@ipoporto.min-saude.pt (V.M.-G.); 2 Department of Pathology, Portuguese Oncology Institute of Porto (IPOP), R. Dr. António Bernardino de Almeida, 4200-072 Porto, Portugal; 3 Department of Pathology and Molecular Immunology, Institute of Biomedical Sciences Abel Salazar, University of Porto (ICBAS-UP), Rua Jorge Viterbo Ferreira 228, 4050-513 Porto, Portugal; 4 Princess Máxima Center for Pediatric Oncology, Heidelberglaan 25, 3584 CS Utrecht, The Netherlands; l.looijenga@prinsesmaximacentrum.nl; 5 Master in Oncology, Institute of Biomedical Sciences Abel Salazar, University of Porto (ICBAS-UP), Rua Jorge Viterbo Ferreira 228, 4050-513 Porto, Portugal; 6 Institut des Biomolécules Max Mousseron (IBMM), CNRS, Université de Montpellier, ENSCM UMR 5247, 34296 Montpellier, France; marie.lopez@cnrs.fr; 7 Epigenetic Chemical Biology, Institut Pasteur, CNRS UMR3523, 75724 Paris, France; paola.arimondo@cnrs.fr

**Keywords:** epidrugs, testicular germ cell tumors, DNA methylation, cell differentiation, drug design

## Abstract

Less toxic treatment strategies for testicular germ cell tumor (TGCT) patients are needed, as overtreatment is a concern due to the long-term side effects of platin-based chemotherapy. Although clinical benefit from classical hypomethylating agents has to date been limited, TGCTs show an abnormal DNA methylome indicating the potential of treating TGCTs with hypomethylating drugs. We tested, for the first time in TGCT cell lines, a new synthetic flavonoid compound (MLo1302) from the 3-nitroflavanone family of DNA methyltransferase (DNMT) inhibitors. We show that MLo1302 reduces cell viability (including of cisplatin resistant cell line NCCIT-R), with IC_50_s (inhibitory concentration 50) within the nanomolar range for NCCIT and NTERA-2 cells, and proved its cytotoxic effect. Exposure to MLo1302 reduced DNMT protein expression, similar to decitabine, and showed a partial effect in cell differentiation, reducing protein expression of pluripotency markers. RT^2^ profiler expression array indicated several dysregulated targets, related to activation of apoptosis, differentiation, and cell cycle arrest. We validated these data by showing increased apoptosis, increased protein expression of cleaved caspase 8 and activated caspase 2, and reduced proliferation (BrdU assay), with increase in CDKN1A and decrease in MIB-1 expression. Therefore, synthetic drugs designed to target DNA methylation in cells may uncover effective treatments for TGCT patients.

## 1. Introduction

Testicular germ cell tumors (TGCTs) are among the most common solid cancers in young-adult men of Caucasian descent, and incidence is increasing worldwide due to lifestyle-related factors [1]. Survival rates are high (overall above 90–95%), but overtreatment of indolent disease that would not recur is an emerging and growing concern, especially due to the long-term side effects of platin-based chemotherapy, affecting quality of life of very young patients [2]. Novel therapies that are less toxic or combination approaches that allow dose reduction are, thus, desirable. This is particularly relevant in the subset of patients developing cisplatin resistance, as no effective treatment options for targeting this poor prognosis disease phenotype exist [3,4].

The molecular background and the morphology of TGCTs are very rich. The most common TGCTs (type II) derive from the common precursor lesion germ cell neoplasia in situ, and are categorized as seminomas or non-seminomas, the latter including embryonal carcinoma, yolk sac tumor, choriocarcinoma, teratoma and mixed tumors, containing at least two or more of these elements. This tumor model resembles embryonic and germ cell development, where epigenetic changes are critical events [5]. Since these tumors are epigenetically distinct, and epigenetics has been demonstrated to contribute to treatment resistance, “epidrugs” present themselves as good candidates for targeting TGCTs [6].

DNA methylation is one of the most well-known epigenetic mechanisms regulating gene expression and is catalyzed by DNA methyltransferases (DNMTs), leading to gene repression (namely of tumor suppressor genes within cancer cells) when occurring on gene promoters [7]. DNMT inhibitors, such as 5-azacitidine (5-AZA) and decitabine (DAC), are approved and have been used successfully for treating hematological malignancies [8], showing promising in vitro and in vivo results in solid neoplasms [9]. In TGCTs, despite demonstrating activity in pre-clinical studies [10,11], results in clinical trials have been less satisfactory due to limited efficacy or important side effects [12,13].

In this study, we aimed to test the in vitro activity of a newly synthesized chemical compound (MLo1302) belonging to the 3-nitroflavanone family of compounds, engineered to inhibit DNMTs in cells. The compound corresponds to “compound 1” in reference [14], where chemical synthesis and structure are fully detailed. Briefly, the substitution of 3-position of the 3-chloro-3-nitroflavanones was modulated, for the preparation of this 3-methyl-3-nitro-substituted compound. We have picked this compound based on the good results obtained upon testing it on renal cell tumor cell lines in our group (ongoing study). Specifically, we sought to determine its ability to induce cell differentiation (comparing it with all-trans retinoic acid—ATRA) and, importantly, to inhibit DNA methylation (comparing with DAC); and to better understand the pathways involved in its mechanism of action.

## 2. Materials and Methods

### 2.1. Cell Lines

TGCT cell lines (NCCIT, 2102Ep, and NTERA-2) were provided by Prof. Leendert Looijenga, and cultured as described [5]. Additionally, a cisplatin resistant clone of NCCIT (NCCIT-R) (kindly provided by Prof. Daniel Nettersheim and generated by Dr. Christoph Oing and Prof. Friedemann Honecker) was included. Cisplatin resistance was validated and this clone was obtained through prolonged culturing under sublethal doses of cisplatin (fully described in [15]).

NCCIT (p53 mutated) is representative of a primary mediastinal mixed non-seminoma with embryonal carcinoma; 2102Ep is representative of a primary testicular mixed non-seminoma with embryonal carcinoma, p53 wild-type; and NTERA-2 is representative of a primary testicular embryonal carcinoma, p53 wild-type. Cells were maintained in low passages and were confirmed negative for Mycoplasma spp. (Clontech Laboratories; Mountain View, CA, USA; twice a month test).

The cell lines used in this study were authenticated via STR profiling, with profiles compared to the database https://www.lgcstandards-atcc.org/STR%20Database.aspx?geo_country=pt and to the ones available in previous publications [16,17]. All experiments included a minimum of three biological replicates, each with experimental triplicates. This study was approved by the Ethics Committee (CES-IPO-12-018, 11 January 2018) of Portuguese Oncology Institute of Porto, Portugal.

### 2.2. Drugs, Cell Viability, and IC_50_

The three cell lines were treated with: ATRA (10 µM, STEMCELL^TM^ Technologies, Vancouver, BC, Canada) for 10 days (with drug renewal every two days); DAC (10 nM–5 µM, Sigma-Aldrich, St. Louis, MO, USA) for 72 h (with drug renewal daily) and MLo1302 (500 nM–10 µM, designed as previously described [14]), for 72 h (with drug renewal daily), after which DNA, RNA, and protein were extracted (see below). Cell morphology was also assessed after treatment with the drugs, and cell perimeter, area, and sphericity were measured.

Cell viability was assessed at 24 h, 48 h, and 72 h of treatment with DAC and MLo1302, as described in [15]. Cells were plated at density of 8000, 6000, and 4000 cells/well (for NCCIT, 2102EP, and NTERA-2, respectively), incubated with Resazurin (Canvax Biotech, Córdoba, Spain) and quantification was performed in a microplate reader (560 nm) (Fluostar Omega, BMG Labtech, Ortenberg, Germany). Optical density (OD) values were corrected to Resazurin-only wells and normalized for day zero. IC_50_ values were computed as described [15].

### 2.3. Apoptosis Assay

Apoptosis assay was performed at 72 h of treatment with MLo1302 according to the protocol detailed in [15], using the Cell-APOPercentageTM apoptosis assay kit (Biocolor, Carrickfergus, UK). For this assay, cells were seeded in 24-well plates at a density of 40,000, 30,000, and 35,000 cells/well (for NCCIT, 2102EP, and NTERA-2, respectively). Quantification was performed by colorimetry (550 nm). The positive control of the reaction was H_2_O_2_. The OD obtained for apoptosis assay was normalized for the OD obtained by viability assay at the same time point.

### 2.4. Proliferation Assay

For assessing cell proliferation, BrdU assay (Cell Proliferation ELISA 5-bromo-2′-deoxyuridine) was performed 72 h after treatment with MLo1302, following the protocol fully described in [15]. Density of cells plated for this assay was of 8000, 6000, and 4000 cells/well (for NCCIT, 2102EP, and NTERA-2, respectively). After previous incubation with BrdU labeling solution (for 12 h), cells were fixed and incubated with anti-BrdU-POD antibody. Quantification was achieved in a microplate reader reaction (450 nm). ODs were normalized to day zero.

### 2.5. Lactate Dehydrogenase (LDH) Release Assay

The cytotoxic effect of MLo1302 compound was evaluated through LDH activity in supernatant cell culture medium by NADH kinetics function. Briefly, TGCT cells were plated at desired density in 96 well plate and allowed to adhere overnight at 37 °C, 5% CO2. Then, TGCT cells were treated with 1/2IC_50_, IC_50_, and 2 × IC_50_ values every 24 h until 72 h. The supernatants were collected every 24 h before MLo1302 treatment renewal and stored at −20 °C. For LDH quantification, samples were incubated with 0.21 mM β-NADH in 0.05 M phosphate buffer at 30 °C for 5 min, followed 23 mM sodium pyruvate. The kinetics of NADH disappearance was followed at 340 nm during 3 min at 30 °C. The levels of LDH released were normalized to positive control and to the number of live cells. LDH levels in MLo1302 conditions were normalized for control condition.

### 2.6. DNA Extraction, Bisulfite Treatment, and Quantitative Methylation-Specific PCR (qMSP)

DNA was extracted from cell lines using phenol-chloroform method, quantified in NanoDropTM Lite Spectophotometer (Cat. ND-LITE, Thermo ScientificTM) and 500 ng were bisulfite-treated using the EZ-96 DNA Methylation-Gold Kit (Zymo Research, Tustin, CA, USA), according to manufacturers’ protocol. qMSP reactions were run in a 7500 Real-Time PCR System (Applied Biosystems^®^, Foster City, CA, USA) as reported in [18], using specific primers (Supplementary Table S1) for *RASSF1A* gene promoter (previously shown to be a tissue biomarker of TGCTs [19]). Results were normalized to ACTB as previously described [18], and computed using the ΔΔCt method. Reactions were run in biological triplicates, each with experimental triplicates. No template, positive (CpGenome Universal Methylated DNA, Millipore) and negative (EpiScope Unmethylated HCT116 DKI gDNA, ref. 3521, TAKARA) controls were included in every plate.

### 2.7. RNA Extraction and Real-Time Quantitative PCR (RT-qCR)

Total RNA was extracted using TRIzol method, quantified and reverse transcribed as reported in [20]. RT-qPCR was performed with master mixes and conditions reported in [20,21] and run in QuantStudio Flex12 for CDKN1A and MIB-1 using specific TaqMan expression assay and primers, normalized to GUSB (Supplementary Table S1). Results were computed using ΔΔCt method and plotted as fold change compared to untreated control. Reactions were performed in five biological replicates, in triplicates.

### 2.8. Western Blot

Western blot was performed according to the protocol detailed in [15]. Proteins were loaded in 8%/12.5% polyacrylamide gels (as appropriate). Blocking of nitrocellulose membranes (Bio-Rad, Germany) was done for 1h at room temperature with 5% dry milk. Primary antibodies are discriminated in Supplementary Table S2, and were incubated at 4 °C. Bands were assessed by chemiluminescence and quantified in Image J (National Institutes of Health, version 1.6.1, 2010), normalized to ACTB.

### 2.9. Immunofluorescence

Immunofluorescence for 5-methylcytosine (5 mC) was performed after 72 h of MLo1302 treatment. Cells were seeded in dark 96-well plates at 4000, 3000, and 2000 cells/well density (for NCCIT, 2102EP and NTERA-2, respectively). Cells were fixed with 4% paraformaldehyde for 15 min and permeabilized with 0.5% Triton X solution in 0.5% Tween-20 for 20 min. Before blocking with 5% bovine serum albumin 1 h at room temperature, cells were treated with 4M HCl during 20 min following trypsin at 37 °C for 2 min. Then, cells were incubated with primary antibody for 5 mC (Millipore, 1:100) (Supplementary Table S2), overnight at 4 °C. In the next day, the cells were incubated with secondary antibody anti-mouse Alexa Fluor 594 (Molecular Probes, Invitrogen, Carlsbad, CA, USA), for 1 h at room temperature. Subsequently, cells were stained with 4′,6′-diamidino-2-phenylindole (1:5 dilution, AR1176, BOSTER Biological Technologies, Beijing, China). Pictures were taken in a fluorescence microscope Olympus IX51 with a digital camera Olympus XM10 using CellSens software (Olympus, Tokyo, Japan). For quantification, in acquired pictures, mean fluorescence intensity per cells was assessed by ImageJ software (version 1.6.1, National Institutes of Health) and normalized for controls of each respective condition.

Additionally, NCCIT cells were incubated with anti-5hmC antibody and the same protocol was followed, using secondary antibody anti-rabbit Alexa Fluor 488 (Molecular Probes, Invitrogen, Carlsbad, CA, USA).

### 2.10. Dot Blot

DNA was extracted and quantified as mentioned before. One thousand nanograms of DNA were diluted in TE buffer. Subsequently, DNA was denatured in 0.1 M of NaOH at 95 °C for 10 min and single chains were stabilized in 1 M of ammonium acetate. DNA was pipetted into nitrocellulose membranes, which were allowed to dry for 30 min at 37 °C. Membranes were exposed to UV light for 1 min to produce crosslinks between DNA and membranes. Then, membranes were blocked with 5% dry milk in TBS with 0.1% Tween and incubated with primary antibody against 5mC (Millipore, 1:1000) at 4 °C, overnight. On the next day, membranes were incubated with secondary horseradish peroxidase conjugated antibody (Cell Signaling Technology) anti-mouse (1:5000), for 1 h at room temperature. As for western blot, the blots were detected using enhanced chemiluminescence and quantified using ImageJ software. Sybergreen I nucleic acid stain (Molecular Probes, 57567, Invitrogen, Carlsbad, CA, USA) was used as loading control.

### 2.11. RT^2^ Profiler Array

Total RNA was extracted from NCCIT cell line (four biological replicates) using TRIzol (Invitrogen, Carlsbad, CA, USA) and quantified in NanoDropTM Lite Spectophotometer (Cat. ND-LITE, Thermo ScientificTM, Waltham, MA, USA). Four hundred nanograms of cDNA were synthetized using Transcriptor High Fidelity cDNA Synthesis Kit (Qiagen, Hilden, Germany), according to manufacturer’s instructions. The RT^2^ Profiler PCR Array System Kit (Qiagen, Hilden, Germany) included 96 genes corresponding to cancer research molecular pathways and adequate controls, in quadruplicates. The expression levels were determined by real-time quantitative PCR in a LightCycler 48 platform (Roche Diagnostics, Risch-Rotkreuz, Switzerland) and ACTINβ, β2M, GAPDH, HPRT1, and RPLP0 were used as endogenous controls. The RT^2^ profiler PCR array analysis was performed in Qiagen specific platform. The data analysis in web portal calculates fold change using ΔΔCt method. Genes with a logarithm fold change above 1.5 or below −1.5 were considered. Additionally, DNA genomic contamination, as well as first strand synthesis and real-time PCR efficiency were monitored in Qiagen platform for RT^2^ profiler PCR array analysis. The lower limit detection was set at Ct ≥ 35.

### 2.12. Statistical Analysis

Data was tabulated using Microsoft Excel 2016 and analyzed using GraphPad Prism 6. Nonparametric tests (Mann-Whitney and Kruskal-Wallis tests) were used for comparing levels among samples. All *p*-values were adjusted for multiple comparisons (Dunn’s test). Statistical significance was set at *p* < 0.05 and is reported in graphs/figures as such: * *p* < 0.05; ** *p* < 0.01; *** *p* < 0.001; **** *p* < 0.0001.

## 3. Results

### 3.1. MLo1302 Reduces Tumor Cell Viability Across TGCT Cell Lines

**Figure 1:**
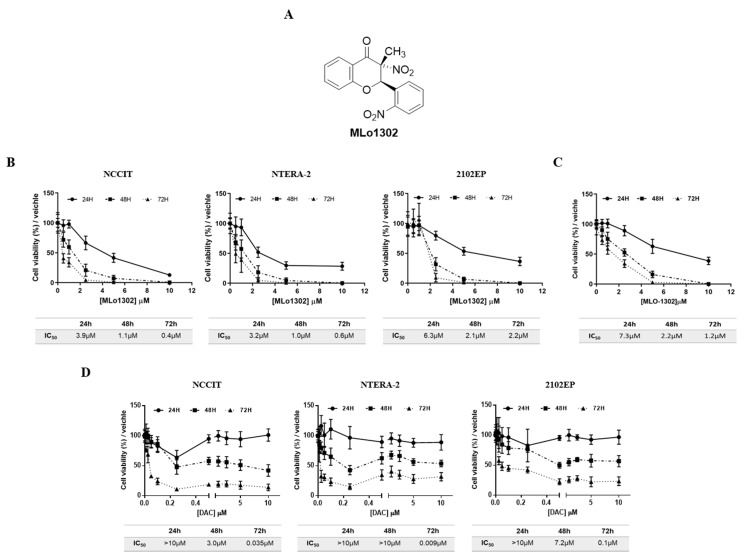
Chemical structure of MLo1302 (**A**) and viability plots corresponding to treatment with MLo1302 in cisplatin sensitive cell lines NCCIT, NTERA-2, and 2102EP (**B**) and in cisplatin resistant NCCIT-R (**C**), and also with DAC (**D**) at 24 h, 48 h, and 72 h of treatment. Respective IC_50_s are represented below each graph.

An illustration of the MLo1302 chemical structure is included in Figure 1A. Treatment with MLo1302 induced a decrease in cell viability in NCCIT, NTERA-2, and 2102Ep cell lines. The effect was dose-dependent, for all time points, and zero percent viability was achieved for all cell lines. Moreover, cell viability was dependent on time of exposure to the drug, being the lowest after 72 h of exposure. The most sensitive cell lines were NCCIT and NTERA-2 (IC_50_ of 400 nM and 600 nM, respectively), followed by 2102EP (IC_50_ of 2.2 µM) (Figure 1B). Additionally, MLo1302 also decreased viability in NCCIT-R cell line, achieving an IC_50_ of 1.2µM (Figure 1C).

Regarding DAC treatment (Figure 1D), cell viability also decreased in all cell lines with nanomolar doses of DAC until around 10–30% viability after 72 h exposure. Again, the effect was more pronounced in NCCIT and NTERA-2, and less expressive in 2102Ep cells. Concentrations above 0.25 µM did not produce additional effect on viability. The effect was also dependent on time, with lower viability at 72 h of exposure (IC_50_ values of 35 nM for NCCIT, 9 nM for NTERA-2, and 100 nM for 2102EP).

The concentrations of 10 nM (previously showed to have antitumor effect in TGCT cell lines [22]) and 1 µM (shown to induce differentiation in somatic tumors [23]) DAC, and of 500 nM (close to the IC_50_ for NCCIT/NTERA-2 at 72 h), 250 nM (1/2IC_50_) and 1 µM (2IC_50_) of MLo1302 were selected to perform the phenotypical and molecular assays (see below).

### 3.2. MLo1302 Treatment Results in Increased Apoptosis and Cytotoxicity, Decreased Cell Proliferation, and Reduces the Protein Expression of Pluripotency Factors

**Figure 2:**
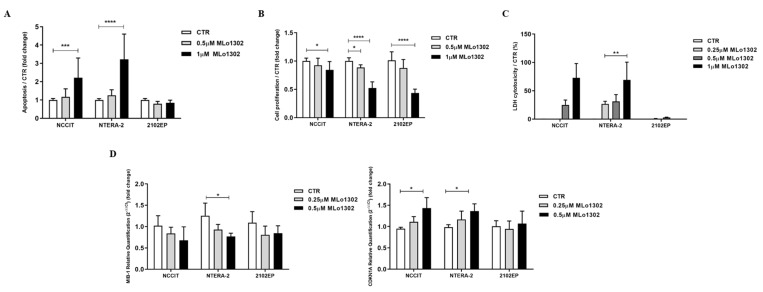
Phenotypical effect of MLo1302. (**A**) Apoptosis assay in cell lines treated with MLo1302 for 72 h; (**B**) Proliferation assay (BrdU) in cell lines treated with MLo1302 for 72 h; (**C**) LDH assay for cytotoxicity in cell lines treated with MLo1302; (**D**) Real-Time Quantitative PCR (RT-qPCR) for MIB-1 and CDKN1A in MLo1302 treated cell lines. * *p* < 0.05; ** *p* < 0.01; *** *p* < 0.001; **** *p* < 0.0001.

We found a significant increase in apoptosis levels in treated NCCIT and NTERA-2 cell lines (Figure 2A) at 72 h of exposure to 1 µM MLo1302, whereas no significant effect was apparent in 2102EP.

Moreover, we found that exposure to MLo1302 for 72 h induced a significant decrease in cell proliferation in all cell lines (Figure 2B). MLo1302 treatment induced an increase in LDH release to the conditioned medium, in NCCIT and NTERA-2 cell lines, and was more prominent with 1 µM concentration (Figure 2C), demonstrating cytotoxicity. However, this was not observed for 2102EP (the cell line also exhibiting less effect on cell viability, see above).

This was accompanied by a decrease in MIB-1 transcript levels (statistically significant in NTERA-2 with 500 nM) and by an increase in transcript levels of CDKN1A, indicating cell cycle arrest (statistically significant for NCCIT and NTERA-2 with 500 nM) (Figure 2D).

ATRA classically induces differentiation of human germ cell tumor lines, which is phenotypically appreciated based on change of morphology (neuronal-like changes) [24]. DNMT inhibitors were shown to produce changes in differentiation at the molecular level, downregulating pluripotency factors in TGCT cell lines [22]. We aimed to determine whether MLo1302 might also produce such effect, either at morphological or molecular level.

**Figure 3:**
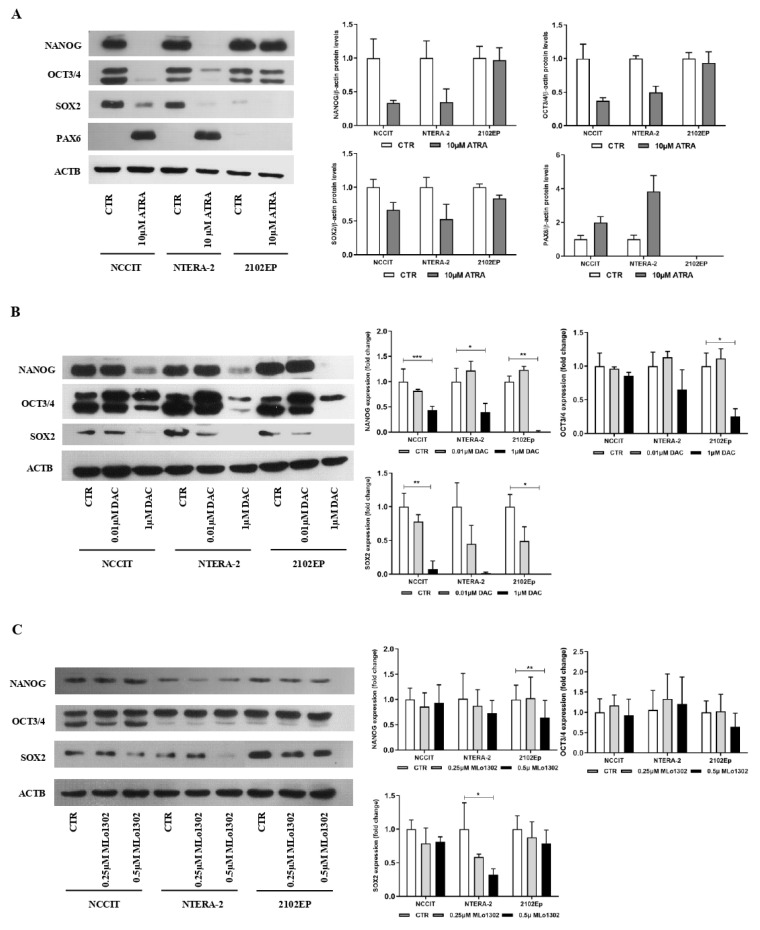
Effect of the several compounds on pluripotency factors. Western blot results for pluripotency and differentiation markers in cell lines, treated with (**A**) ATRA, (**B**) DAC; and (**C**) MLo1302, at the indicated concentration. Beta-actin is used as normalizer. Band densitometry graphs are presented (treated versus control). * *p* < 0.05; ** *p* < 0.01; *** *p* < 0.001.

As described, ATRA induced differentiation in NCCIT and NTERA-2 cell lines. Morphometric analysis of the cells with most prominent and sustained phenotypical changes (NTERA-2) showed a statistically significant increase in cell perimeter, and decrease in area and sphericity, approximating a neuronal shape (Supplementary Figure S1). These phenotypical changes were accompanied by a decrease in pluripotency-related markers (and an increase in PAX6) at protein level, more evident for NCCIT and NTERA-2 (Figure 3A). For both DAC and MLo1302, no effect was seen at the morphological level in any of the cell lines in the study, which maintained the same morphology.

We then evaluated effect on differentiation at the molecular level, by assessing protein levels of pluripotency factors. Regarding DAC treatment, the concentration of 10 nM decreased pluripotency factors (especially SOX2), and the higher concentration of 1 µM produced a more pronounced effect across the cell lines for SOX2, NANOG, and OCT3/4 (Figure 3B). However, concerning MLo1302, the lower dose of 250 nM had little effect on cell differentiation, while the higher dose of 500 nM led to significant decrease in NANOG (in 2102EP and NTERA-2) and in SOX2 (in NTERA-2) protein expression (Figure 3C).

### 3.3. MLo1302 Effect on DNMTs, Global DNA Methylation (5 mC), and Loci-Specific Methylation Status

Upon ATRA-induced differentiation a significant decrease in DNMT3B expression was verified (as reported elsewhere [25]), with no significant changes in expression of other DNMTs (Supplementary Figure S2A). Moreover, we found increased levels of *RASSF1A* promoter methylation in differentiated NCCIT and NTERA-2 cells (Supplementary Figure S3A). DAC confirmed its hypomethylating effect, producing an overall decrease in DNMT expression with the higher concentration of 1 µM (but not significant with the 10 nM dose, Supplementary Figure S2B), and a decrease in *RASSF1A* promoter methylation (Supplementary Figure S3B), as previously reported [26]. Although no significant change was seen in global methylation levels, assessed by 5 mC dot blot (Supplementary Figure S2C), a decrease in 5 mC levels assessed by immunofluorescence was confirmed (Supplementary Figure S2D).

**Figure 4:**
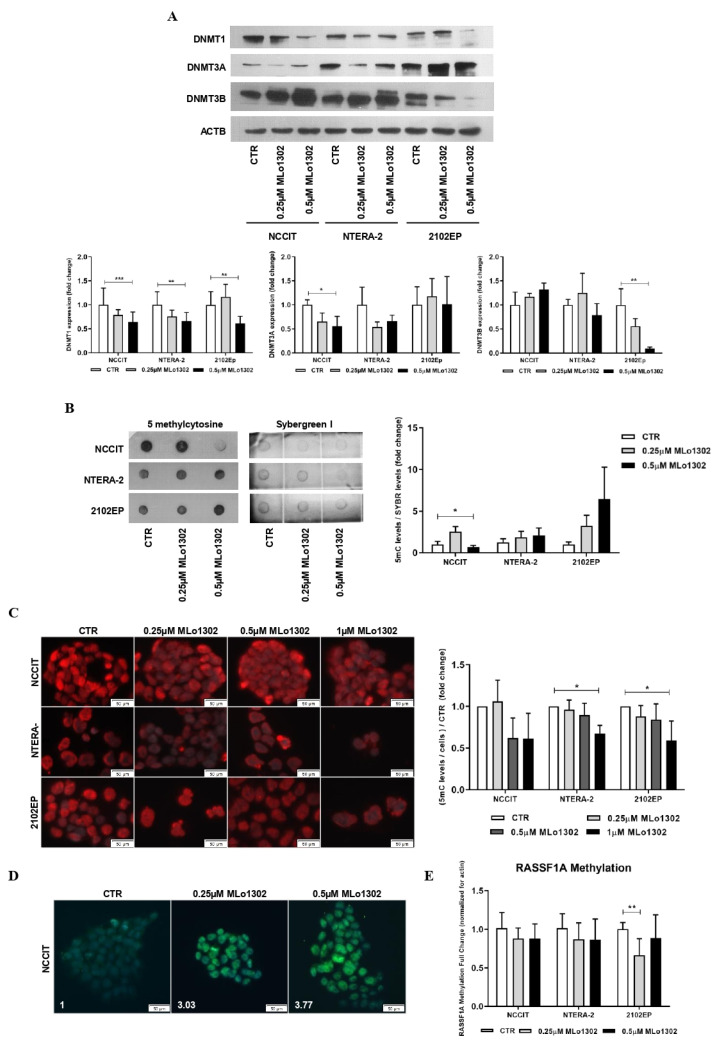
Effect of MLo1302 treatment on DNA methyltransferases (DNMTs) expression and on DNA methylation profile. (**A**) Western blot for DNMTs in cell lines treated with MLo1302. Beta-actin is used as normalizer. Band densitometry graphs are provided (treated versus control); (**B**) Dotblot for 5 mC in cell lines treated with MLo1302, and respective quantification. Results are normalized to Sybergreen; (**C**) Immunofluorescence for 5 mC in cell lines treated with MLo1302, and respective quantification; (**D**) Immunofluorescence for 5 hmC in NCCIT cell line treated with MLo1302; (**E**) *RASSF1A* promoter DNA methylation in cell lines treated with MLo1302. Results are normalized to beta-actin. * *p* < 0.05; ** *p* < 0.01; *** *p* < 0.001.

Concerning MLo1302, the lower concentration of 250 nM produced a decrease of DNMT3A expression in NCCIT and NTERA-2 and of DNMT3B in 2102EP cells, but more remarkable and statistically significant effects were depicted for the higher dose of 500 nM for DNMT1 expression (Figure 4A). Additionally, using dot blot analysis, we observed at 500 nM treatment a significant decrease in 5 mC global levels in NCCIT cells, but not in NTERA-2 or 2102EP (Figure 4B). Nonetheless, decreased 5 mC global levels were confirmed in all cell lines using immunofluorescence (Figure 4C; further figures illustrative of the effect on cell death are presented in Supplementary Figure S4). Moreover, and since DAC has been shown to induce a paradoxical increase in 5hmC levels thanks to a partial selectivity of TET enzymes to hemi-methylated CpGs [27], we aimed to test this hypothesis with MLo1302. Indeed, we found an increase of 5 hmC levels in NCCIT-treated cells (Figure 4D). Despite a decrease in DNA methylation of *RASSF1A* promoter was detected for 2102EP, no significant effect was achieved in the remaining two cell lines (Figure 4E).

### 3.4. Molecular Characterization of Pathways Deregulated by MLo1302 Treatment

**Figure 5:**
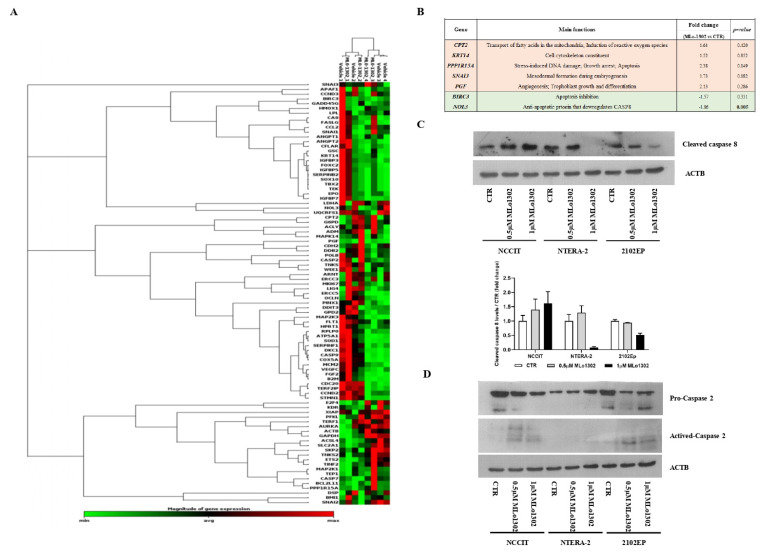
Downstream pathways activated by MLo1302. (**A**) RT^2^ profiler expression array for cell lines treated with MLo1302. Targets with fold change (treated versus control) over 1.5 are shown, together with *p*-value (**B**); (**C**) Western blot for cleaved caspase 8 in cell lines treated with MLo1302. Beta-actin is used as normalizer. Band densitometry graph is provided below (treated versus control); (**D**) Western blot for activated caspase 2 in cell lines treated with MLo1302. Beta-actin is used as normalizer.

Next, we assessed which targets/pathways are deregulated upon treatment with the compound using the RT^2^ profiler array (Figure 5A). The array identified *NOL3* as the most significantly downregulated target (fold change −1.86, *p* = 0.005, Figure 5B). *NOL3* is an antiapoptotic player, known to inhibit various caspase isoforms, including caspase 8 and 2 [28,29]. We confirmed the increase of cleaved caspase 8 protein expression by treating NCCIT cell line with 0.5 µM of MLo1302 (Figure 5C). We also found increase in protein expression of activated caspase 2 in NCCIT and 2102EP, with no change in procaspase 2 expression (Figure 5D). Other targets found to be upregulated include *CPT2* (involved in fatty-acids beta-oxidation and generation of reactive oxygen species, which have a role in promoting apoptosis [30]), *SNAI3* (related to embryogenesis and mesodermal differentiation, and found to be expressed in germ cell tumors [31]), *PGF* (related to trophoblast differentiation [32]), and *PPP1R15A* (related to stress-induced DNA damage, growth arrest and apoptosis [33]). Additionally, *BIRC3*, another inhibitor of apoptosis, involved in therapy resistance, was downregulated [34].

## 4. Discussion

Novel treatment options for TGCT patients, less toxic than the classical (although highly effective) cisplatin-based chemotherapy are needed. These new therapies may allow dose reduction, sparing very young patients from the undesirable side effects of chemotherapeutic drugs, which may severely affect their quality of life [2,35]. These new drugs become even more relevant in the context of cisplatin resistance, since no effective treatments exist for these patients, entailing poor prognosis [36,37,38]. Epidrugs have been tested in TGCTs (for recent reviews see [6,13]), and despite good pre-clinical (in vitro and in vivo) results, achieving antitumor effect with low-range doses and little toxicity, patient trials have not been successful. This may be due to the very poor health status of selected (i.e., resistant) patients; not achieving the most beneficial dose, causing the least side effects; or very likely not finding the optimal combination of drugs for simultaneously and effectively targeting several cancer hallmarks [39]. To date, the most explored epidrugs have been nucleoside DNMT inhibitors [10,11,22,40], such as 5-AZA and DAC, already approved for treatment of hematological malignancies [8]. Although DNMTs were found to be differentially expressed in TGCTs [20], these nucleoside DNMT inhibitors were more limited in producing clinical benefit (although a recent trial using guadecitabine, the prodrug of DAC, in combination with cisplatin in metastatic platinum-refractory germ cell tumor patients produced promising results, with the combination being well tolerated and achieving a clinical benefit rate of 46% [41]). Herein, we aimed to take advantage of a newly synthesized flavanone derivative (MLo1302) from the 3-nitroflavanone DNMT inhibitor family [14], and to assess its mechanisms of action in TGCT cell lines.

Remarkably, MLo1302 reduced cell viability in three non-seminoma cell lines, with IC_50_ values in the nanomolar range, for NCCIT and NTERA-2 cell lines. The optimal dose of DAC for treating patients in the clinic is still under debate, as is the schedule of administration, another very relevant variable [42,43]. In the latest clinical trial [41], guadecitabine was given to TGCT patients on days 1 to 5 by subcutaneous injection and the maximal tolerated dose of 30 mg/m^2^ was established. Considering an average 70 kg male, this corresponds to an approximate 100 uM concentration of the drug, so we hypothesize that MLo1302 concentrations used in our work may be effective and nontoxic in the clinic. 2102EP cell line was the least sensitive to treatment, also observed upon exposure to DAC. For both drugs, the effect was dependent on time of exposure and concentration; overall, at 72 h, IC_50_ values were lower for DAC compared to MLo1302. With DAC treatment, however, we found no additional effect on viability beyond the dose of 250 nM, even upon exposure to very high doses (e.g., 10 µM). The surviving cells may endure the exposure to the drug by activating cell differentiation, as demonstrated by downregulation of pluripotency factors upon exposure to high doses of DAC. On the contrary, exposure to high doses of MLo1302 reduced viability to zero in all cell lines. Interestingly, Albany et al. [22] obtained IC_50_ values in the same range (IC_50_ of 10 nM in NTERA-2 cell line, for instance) upon exposure to DAC. These values reflect a higher sensitivity of TGCTs to this family of compounds, rendering lower IC_50_ values than those observed for many other solid tumors [44]. This is also in line with our recent findings concerning MLo1302 treatment of renal cell carcinoma cell lines, obtaining IC_50_ values in higher ranges than those observed with TGCT cell lines. Importantly, MLo1302 also reduced cell viability in a cisplatin-resistant cell line (NCCIT-R), with an IC_50_ of 1.2 µM. This cell line shows a much higher IC_50_ to cisplatin (31.59 µM), which is the mainstay available drug in the clinic. Further studies should be pursued in the context of cisplatin resistance to support that this compound can also be clinically useful in this context.

In consonance with viability, functional cell assays demonstrated both cytotoxicity (with increased NAD^+^ consumption and LDH release to the cell media) and increased apoptosis in NCCIT and NTERA-2 cell lines, but not in 2102EP, the cell line showing the least pronounced effect in cell viability. However, a significant decrease in cell proliferation was observed for all cell lines upon treatment with MLo1302, and this was supported by increased expression of CDKN1A and decreased MIB-1, indicating cell cycle arrest.

Since hypomethylating agents are known to induce cell differentiation [40], we assessed it for MLo1302 treatment, in comparison with the classical differentiating agent ATRA. As expected, ATRA induced noticeable effects at the morphology of cells, especially in NTERA-2 (as reported), which was accompanied by an abrupt decrease in expression of pluripotency-related markers (*NANOG*, *OCT3/4*, and *SOX2*) and an increase of *PAX6*—(neuronal) differentiation-related marker in NCCIT and NTERA-2 cells. This is less apparent in 2102EP cells, which is less prone to differentiation upon ATRA treatment [17]. Furthermore, DNMT3B showed the same profile as the pluripotency markers, being significantly less expressed in differentiated cells, which is in consonance with previous findings on DNMT3B role, related to stemness and pluripotency [25]. Additionally, effective differentiation with ATRA led to increased *RASSF1A* promoter DNA methylation levels in NCCIT and NTERA-2 cell lines, in line with the reported gradient of increasing global DNA methylation found in these tumors and related to more differentiated histological subtypes [45]. As for DAC and MLo1302, however, no effect on morphology was obtained. These compounds produced, nevertheless, downregulation of pluripotency markers in the higher dose tested, most remarkably for NANOG and SOX2, showing that a partial effect in differentiation is also elicited at the molecular programme.

Treatment with 10 nM DAC was less effective in decreasing DNMT expression, but 1 µM led to a significant decrease of *DNMT1*, *DNMT3A*, and *DNMT3B* protein levels (completely abrogating expression for DNMT1 and DNMT3A). Additionally, we found a decrease in *RASSF1A* promoter methylation after DAC treatment, in line with previous studies [26]. As for MLo1302, our data on TGCTs revealed a significant decrease of *DNMT1* expression in all cell lines, and a decrease of *DNMT3A* and *DNMT3B* expression in some cell lines, when treated with 500 nM. However, the compound had no effect on DNMT3B in NCCIT and NTERA-2 cells, which actually showed increased expression (but not reaching significance). This is in line with results obtained for DAC treatment precisely for NCCIT and NTERA-2 cells (Supplementary Figure S2), where DNMT3B was also the most resistant DNMT to the hypomethylating drug, with less reduced expression compared to complete abrogation of expression for DNMT3A and DNMT1. This may be related to the fundamental role of DNMT3B in these tumors, related to pluripotency. Moreover, it is known that protein expression of epigenetic enzymes does not always reflect their activity, which can be reduced despite maintained protein expression. However, the effect was not consistent and prominent in all cell lines and experiments, as demonstrated by significant decrease in 5 mC levels using dot blot in NCCIT cell line only, and not in NTERA-2 or 2102EP. This is also seen in the study of Jüttermann et al., which indicated that the antitumor effect of DAC is primarily mediated by covalent trapping of DNMTs themselves [46]. In this model, DNMTs bind to DAC-substituted DNA, and this crosslinking is the mediator of the anti-neoplastic effect, meaning that only tumors with high DNMT expression will benefit from those drugs [46]. This might explain the absence of effect on global methylation levels after DAC treatment in dot blot (despite the decrease of DNMT expression). For MLo1302 this covalent trapping of DNMTs is also possible, although less likely since the compound is a non-nucleoside analogue, not incorporated into DNA. Another possibility is that the mechanism of action of this compound is distinct, inducing hypomethylation only in specific regions of the genome, which would explain undetectable differences overall in global methylation. Moreover, we could not detect decrease in methylation of RASSF1A promoter after MLo1302 treatment in NCCIT and NTERA-2 cell lines, which can be due to this more selective region-specific hypomethylating action of the compound or simply due to study of less relevant CpG sites in our study, whose methylation status is not changed after treatment. Further studies are warranted to clarify these hypotheses.

**Figure 6:**
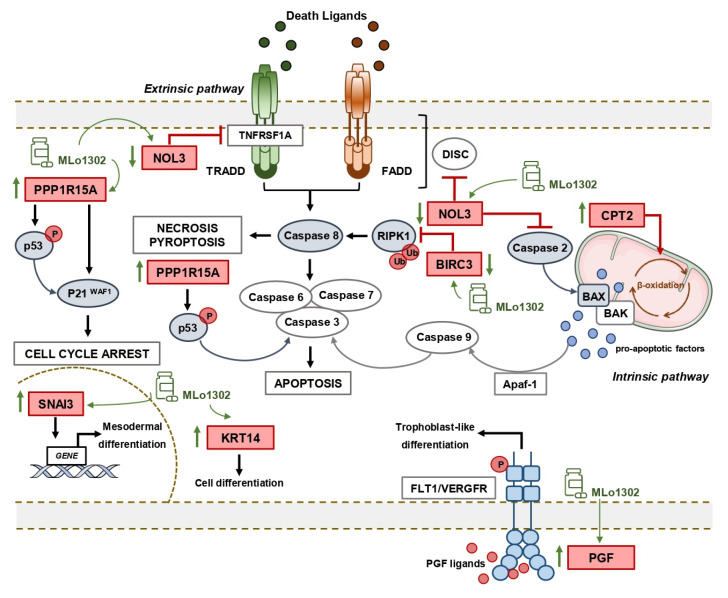
Summary of putative pathways deregulated upon treatment with MLo1302.

We further explored the downstream mechanisms elicited upon MLo1302 treatment. We performed an expression array, which indicated that the apoptotic pathway was the most significantly involved in the drug’s mechanism of action. Seven genes were differentially expressed with fold change above the defined criteria. Specifically, a statistically significant downregulation of the anti-apoptotic gene *NOL3/ARC* was found. *NOL3* is involved in apoptosis and autophagy regulation, by impairing the function of caspase 8 and also of caspase 2 [28,29]. Interestingly, we found that MLo1302 induces higher cleaved caspase 8 protein levels in NCCIT and NTERA-2 and of caspase 2 in NCCIT and 2102EP. NOL3 is also referred to downregulate p53 (which is very relevant in TGCTs, showing a hypersensitive p53 signalling pathway) and on the other hand to be regulated by the p53-induced ubiquitin E3 ligase MDM2 [47]. In fact, this player blocks various modes of cell death by interacting with different pathways: firstly, it inhibits the extrinsic apoptotic pathway, both by interacting with FAS and FADD, blocking death-inducing signalling complex (DISC) assembly and by limiting the amount of *CASP8* available for DISC-mediated activation. Additionally, *NOL3* also blocks the intrinsic apoptotic pathway in response to various stress factors, by inactivating BAX, impeding the release of pro-apoptotic factors by the mitochondria [48]. Moreover, it acts as a cytosolic calcium buffer, impairing calcium-mediated cell death [49]. More specifically, it negatively regulates apoptosis triggered by oxidative damage (by blocking *CASP2* and *BAX* translocation) and induced by hypoxia (by impeding cytochrome c release from mitochondria). Finally, it also interferes with TNF-induced necrosis, blocking this pathway by interacting with *TNFRSF1A* [50]. *CASP8* in particular also has a role in other forms of cell death, like necroptosis and pyroptosis, which can be further explored [51]. Other targets found to be deregulated (although not achieving significance) were again related to apoptosis, but also to cell differentiation (in line with our previous results on pluripotency factors) and cell cycle arrest. In particular, *BIRC3* was also downregulated, and this protein was shown to impair apoptosis by inhibiting ripoptosome formation, by ubiquitinating *RIPK1* and, again, *CASP8*. This player is also a relevant regulator of the NF-kappa-B signalling pathway and also of the innate immune response [52]. Moreover, *PPP1R15A* is activated in response to stress and involved in activation of p53 by phosphorylation of Ser-15 and in activation of p21/WAF1, promoting apoptosis and leading to cell cycle arrest [53]. It is further involved in disturbing the TGF-beta pathway and in promoting apoptosis by activation of *CASP3* [54]. On another perspective, the intermediate filament *KRT14* has been demonstrated to promote squamous cell differentiation in a *NOTCH1*-dependent manner [55]; *SNAI3*, a member of the SNAIL family, is involved in mesodermal differentiation during embryogenesis and regulates epithelial-to-mesenchymal transition [56]; while *PGF* is a key factor in trophoblast differentiation, which binds to the receptor *FLT1/VEGFR-1* and activates stress activated protein kinase (SAPK) pathways, leading to endothelial cell growth and neoangiogenesis typical of this tissue [57]. Of relevance, *CPT2* is transported from the nucleus to the inner membrane of the mitochondria. This carnitine palmitoyltransferase (together with acyl-CoA synthetase and carnitine/acylcarnitine translocase) leads to beta-oxidation of fatty acids. A summary of these signalling pathways is represented in Figure 6.

**Figure 7:**
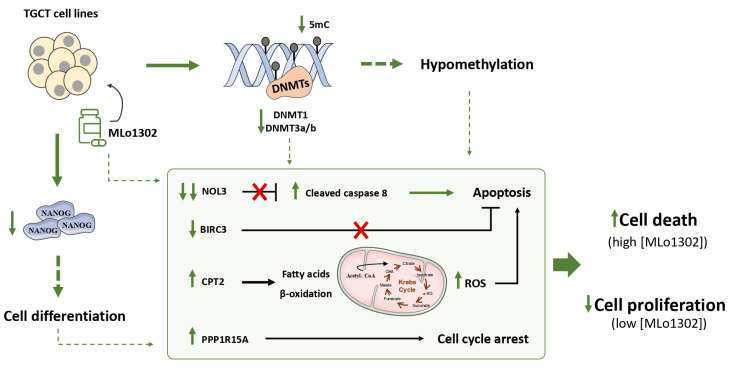
Proposed mechanisms of action of MLo1302. The compound acts by inhibiting DNMTs expression, partially decreasing global DNA methylation levels; induces cell differentiation; and promotes apoptosis and cell cycle arrest.

We explored, for the first time, the effects of a newly synthesized non-nucleoside flavanone compound, in four TGCT cell lines, including a cisplatin resistant clone. DNMT downregulation was observed along with relevant effects on cell differentiation. Importantly, we also demonstrated that MLo1302 cytotoxic effect, is due mostly to stimulation of apoptosis, namely by targeting the anti-apoptotic factor NOL3, and by inducing cell cycle arrest. The effects of MLo1302 may be in part a consequence of DNA hypomethylation, and this may stem indirectly from DNA methylation inhibition or may derive from a non-methylation-dependent effect (Figure 7). An indirect mode of action of MLo1302 leading to a decrease of DNMT expression level is then possible, and identification of MLo1302 direct target(s) could be further investigated in TGCT cell lines via chemical biology approach using affinity-based chemical probes.

## 5. Conclusions

Future studies are needed to definitively answer these questions. One limitation of our work is related to assessment of putative toxicity of the compound in the normal germ cell counterpart, as a measurement of the compound safety. However, to date, no normal germ cell line is available, and cell lines derived from stromal cells do not represent truly the germ cell lineage and react differently to exposure to drugs. In order to answer this important question we intend to assess MLo1302 effect in vivo, using a control group, providing histological and biochemical evidence of the effect in normal subjects. Moreover, MLo1302 may be explored in combinatory strategies, either with cisplatin or other targeted therapies, to improve treatment of TGCT patients. Specifically, following one line of investigation of our group and our previous results [15,58], we intend to combine MLo1302 both with histone deacetylase inhibitors (HDACis) Belinostat and Panobinostat (to assess synergy with hypomethylating properties of MLo1302) and also with anti-PD1/PDL1 agents (to assess the potential for epigenetic activation of tumor microenvironment and enhance response to immunotherapies). Finally, the expression array advances many molecular pathways by which MLo1302 induces cell death (besides downregulation of NOL3 and increase caspase 8 and 2 activation) and these may be explored in future studies.

## Data Availability

All data produced in this work is reported within the manuscript and its supplementary files.

## Supplementary Materials

The following are available online at https://www.mdpi.com/1999-4923/13/1/73/s1, Figure S1: Phenotypical effect of ATRA treatment on NTERA-2 and NCCIT cell lines. (A) Photomicrographs of treated versus control NCCIT cells; (B) Phtomicrographs of treated versus control NTERA-2 cells; (C) Morphometric analysis of NTERA-2 cells; Figure S2: Effect of ATRA and DAC on DNMTs expression and DNA methylation profile. (A) Western blot for DNMTs in cell lines treated with ATRA. Beta-actin is used as normalizer. Band densitometry graph is provided (treated versus control); (B) Western blot for DNMTs in cell lines treated with DAC. Beta-actin is used as normalizer. Band densitometry graph is provided (treated versus control); (C) Dot blot for 5mC in cell lines treated with DAC, and respective quantification. Results are normalized to Sybergreen; (D) Immunofluorescence results for 5mC in DAC-treated cell lines; Figure S3: *RASSF1A* methylation studies in ATRA-(A) and DAC-(B) treated cells. Results are normalized to beta-actin; Figure S4: More representative illustrations of immunofluorescence for 5 mC in MLo1302-treated cell lines, putting in evidence the effect of the compound on cell death. Notice the reduced number of cells in treated condition compared to untreated control; Table S1: Primer sequences used in the work; Table S2: Antibodies used in the work.
