# Supplementary material for: Targeting Germ Cell Tumors with the Newly Synthesized Flavanone-Derived Compound MLo1302 Efficiently Reduces Tumor Cell Viability and Induces Apoptosis and Cell Cycle Arrest
Source: Pharmaceutics. 2021 Jan 7;13(1):73. doi: 10.3390/pharmaceutics13010073 (PMC7826804; doi:10.3390/pharmaceutics13010073)
Supplement: Supplementary file 1 [file pharmaceutics-13-00073-s001.pdf]

pharmaceutics

Supplementary Materials: Targeting Germ Cell Tumors with the Newly Synthesized Flavanone-Derived Compound MLo1302 Efficiently Reduces Tumor Cell Viability and Induces Apoptosis and Cell Cycle Arrest

João Lobo, Ana Rita Cardoso, Vera Miranda-Gonçalves, Leendert H.J. Looijenga, Marie Lopez, Paola B. Arimondo, Rui Henrique and Carmen Jerónimo

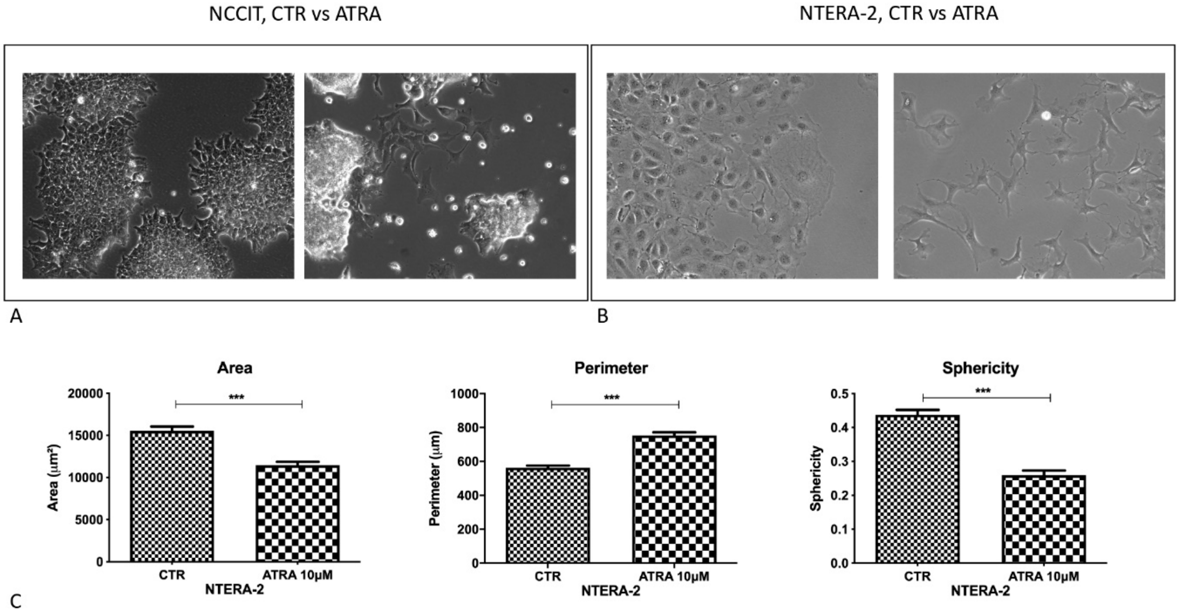

Figure S1. Phenotypical effect of ATRA treatment on NTERA-2 and NCCIT cell lines. **(A)** Photomicrographs of treated versus control NCCIT cells; **(B)** Phtomicrographs of treated versus control NTERA-2 cells; **(C)** Morphometric analysis of NTERA-2 cells.

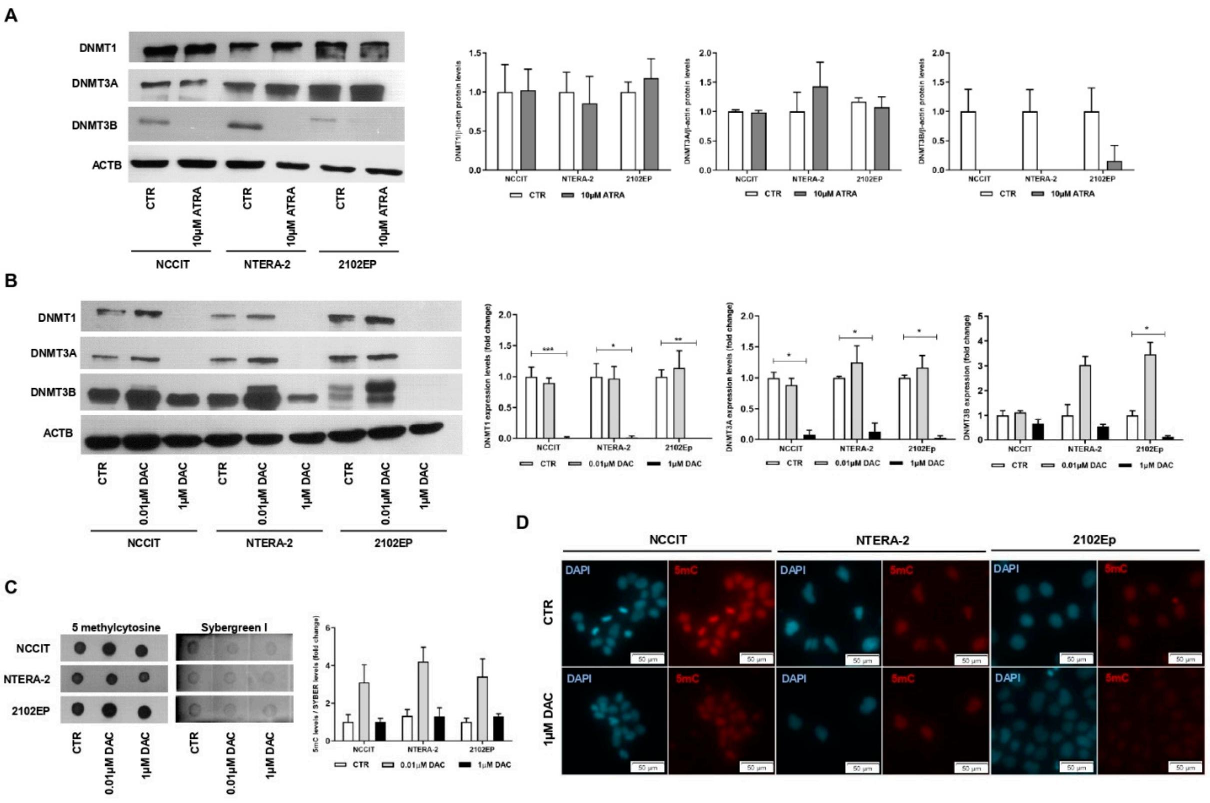

Figure S2. Effect of ATRA and DAC on DNMTs expression and DNA methylation profile. **(A)** Western blot for DNMTs in cell lines treated with ATRA. Beta-actin is used as normalizer. Band densitometry graph is provided (treated versus control); **(B)** Western blot for DNMTs in cell lines treated with DAC. Beta-actin is used as normalizer. Band densitometry graph is provided (treated versus control); **(C)** Dot blot for 5mC in cell lines treated with DAC, and respective quantification. Results are normalized to Sybergreen; **(D)** Immunofluorescence results for 5mC in DAC-treated cell lines.

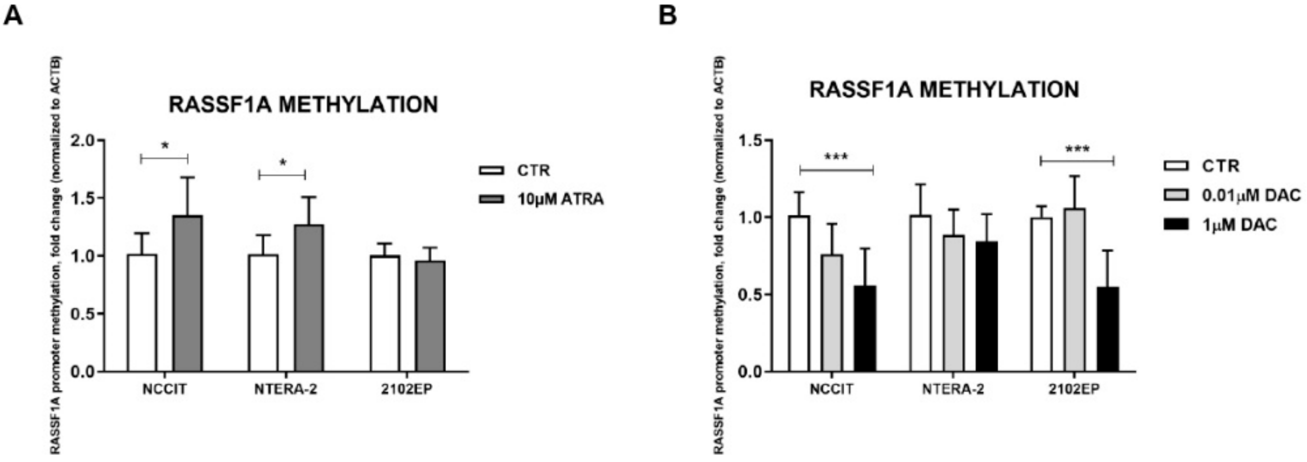

Figure S3. *RASSF1A* methylation studies in ATRA-**(A)** and DAC-**(B)** treated cells. Results are normalized to beta-actin.

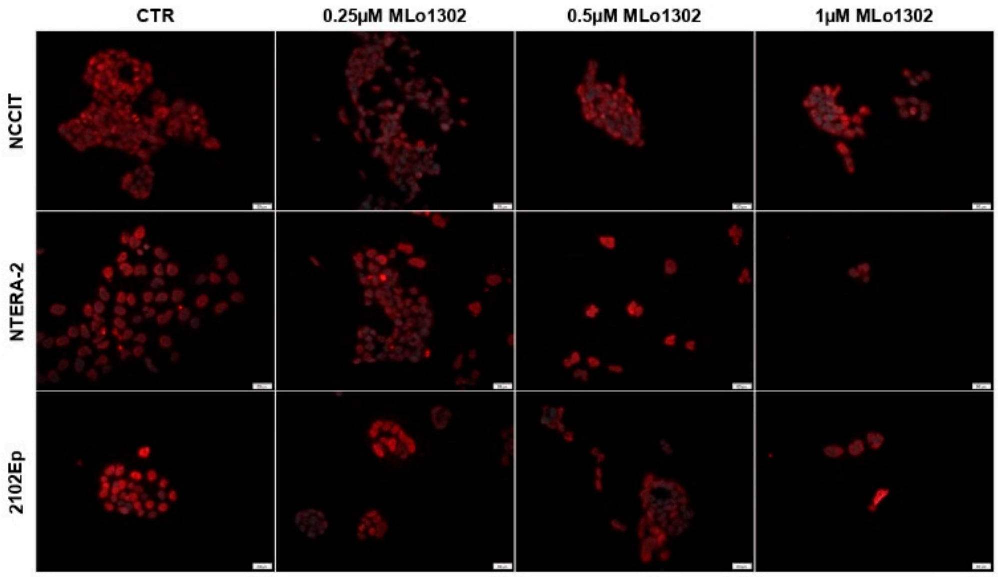

Figure S4. More representative illustrations of immunofluorescence for 5 mC in MLo1302-treated cell lines, putting in evidence the effect of the compound on cell death. Notice the reduced number of cells in treated condition compared to untreated control.

Table S1. Primer sequences used in the work.

Gene	Primer sequence (5′-3′)	10μM primers, volume (μL), F+R	Annealing Temperature (°C)
RASSF1A	F: AGCGAAGTACGGGTTTAATC<br>R: ACACGCTCCAACCGAATA	0.3	60
ACTB	F: TGGTGATGGAGGAGGTTTAGTAAGT<br>R: AACCAATAAAACCTACTCCTCCCTTAA	0.4	60
GUSB	F: CACTGAAGAGTACCAGAAAAGTC<br>R: TCTCTGCCGAGTGAAGATCC	0.5	60
GUSB (TaqMan assay)	Assay ID: Hs99999908	-	60
CDKN1A (TaqMan assay)	Assay ID: Hs00355782	-	60
MIB-1	F: GGACTTTGGGTGCGACTTGA<br>R: ACAACTCTTCCACTGGGACG	0.5	62

Table S2. Antibodies used in the work.

Antibody	Firm/clone	Dilution	Secondary antibody
DNMT1	Cell signaling / D63A6	1:500	Anti-rabbit
DNMT3A	Cell signaling / D23G1	1:250	Anti-rabbit
DNMT3B	Cell signaling / D7070	1:1000	Anti-rabbit
NANOG	Abcam / ab109250	1:1000	Anti-rabbit
OCT3/4	Cell signaling / C52G3	1:1000	Anti-rabbit
SOX2	Cell Marque / SP76	1:250	Anti-rabbit
PAX6	Invitrogen / 13B10-1A10	1:1000	Anti-mouse
Cleaved caspase 8	Cell signaling / D384	1:250	Anti-mouse
Activated caspase 2 / procaspase 2	Cell signaling / #2224	1:250	Anti-mouse
5mC	Calbiochem / 162233D3	1:1000 (dot blot); 1:100 (immunofluorescence)	Anti-mouse
5hmC	Invitrogen / U12848871	1:1000 (immunofluorescence)	Anti-rabbit
ACTB	Sigma-Aldrich / A1978	1:10 000	Anti-mouse
